# Linking socio-economic metabolism models and simulation games: Reflections on benefits and challenges

**DOI:** 10.1111/jiec.13462

**Published:** 2024-02-02

**Authors:** Marta Roca-Puigròs, Andreas Gerber, Markus Ulrich, Matthias Y. Reich, Daniel Beat Müller, Patrick Wäger

**Affiliations:** 1 Empa, Swiss Federal Laboratories for Material Science andTechnology, St. Gallen, CH-9014 Switzerland; 2 Industrial Ecology Programme, Department of Energy and Process Engineering, Norwegian University of Science and Technology (NTNU), Trondheim, Norway; 3 UCS Ulrich Creative Simulations, Uster, Switzerland

**Keywords:** climate change, industrial ecology, interaction tools, role-play, simulation games, socio-economic metabolism

## Abstract

Simulation models of socio-economic metabolisms (SEM) are increasingly used to address questions regarding environmental issues. SEM studies often focus on target audiences such as researchers and decision-makers by means of scientific articles, reports, and oral presentations. In this contribution, we study the linkage of SEM models and simulation games (SGs) (i) to widen the audience of SEM research to include the general public, who, through their lifestyles, contribute to the studied environmental issues, and (ii) to address the different audiences by means of interaction tools. We illustrate the link through a case study, the postfossilCities SG. Based on the case study, existing SGs, and literature, we identified that linking SEM models with SGs can facilitate (1) the accessibility of SEM models to wider audiences, (2) the communication and understanding of systems with complex dynamics, such as nonlinear behavior, through tools that trigger experiences and emotions, (3) the consideration of actors’ dimension of physical systems, and (4) the robustness of SGs by having mass- and energy-balance consistent representations of physical systems. Despite these benefits, linking SEM models and SGs is also bound to challenges, such as (1) the integration of approaches from different disciplines, (2) the high demand on time and financial resources, and (3) the balance between simplification and complexity. In order to facilitate the link between SEM models and SGs, and thus exploit the mentioned benefits, it is important to set-up connections to game-related communities and communities with long tradition of using SGs, and use existing game development methods.

**Supplementary Information:**

The online version of this article (doi:10.1111/jiec.13462) contains supplementary material, which is available to authorized users.

## INTRODUCTION

Simulation models of socio-economic metabolisms (SEM) are increasingly used to address questions regarding environmental issues. For example, SEM models have been used to gain insights into the relationships between greenhouse gas (GHG) emissions and energy and material flows and stocks, through scenarios exploring circular economy and climate change mitigation strategies (Pauliuk et al., 2012; Roca-Puigròs et al., 2023; Streeck et al., 2021). The growing use of SEM models can be attributed—among others—to their multiple and diverse purposes, such as (1) foresight, including forecasting and backcasting, (2) explanation, for example, increase understanding of systems, (3) intervention and optimization, that is, support the design and implementation of system interventions and optimizations, and (4) education, that is, explore the consequences of certain actions (Hilty, 2019; Løvik et al., 2014). While these purposes highlight the relevance and usefulness of SEM studies, SEM studies often focus on target audiences such as policy-makers, industry representatives, and researchers. However, given that most SEM studies address societal problems, a wider target audience could ease the management of such problems.

In this forum article, we propose to link SEM models and simulation games (SGs) to make SEM studies more accessible to a wider audience, and to address the audience by means of interaction tools.

The aim of this forum article is (1) to discuss the value of combining SEM models and SGs and (2) to point out the potential benefits and challenges of linking SEM models and SGs. We pursue these aims, first by (i) describing the current outreach of SEM studies, (ii) reviewing how crucial aspects for the outreach of SEM studies have been tackled in the literature, and (iii) identifying potentials to expand the outreach of SEM studies. Second, we introduce SGs and the simulation models frequently used in SGs addressing environmental issues. Third, we present the case study of the postfossilCities SG to illustrate the link between SEM models and SGs. Fourth, we discuss the potential benefits and challenges of linking SEM models and SGs. Finally, we provide some remarks to support the establishment of a solid link between SEM models and SGs.

## OUTREACH OF SEM STUDIES

SEM studies focus largely on exploring systemic problems, such as climate change and circular economy by analyzing the physical world as a system of stocks and flows based on principles of mass- and energy-balance conservation (Baccini & Brunner, 2012; Fischer-Kowalski, 1998; Fischer-Kowalski & Amann, 2001; Fischer-Kowalski & Haberl, 1998; Pauliuk & Müller, 2014). For example, Streeck et al. (2021) developed a dynamic SEM model representing material stocks in the United States from 1870 to 2100, in order to explore circular economy strategies including increased recycling and limited demand for stocks. Another example is the study of Pauliuk et al. (2021), who used a dynamic SEM model of global passenger vehicles and residential buildings to estimate the potential emissions savings under different scenarios with material efficiency strategies. SEM studies usually target researchers and decision-makers by means of scientific articles, reports, and oral presentations, while largely excluding the general public, who, through their lifestyles and daily choices, contribute to the systemic problems studied in SEM research. Expanding the accessibility of SEM studies to the general public is critical for the success of strategies designed to address complex, systemic problems. In addition, the means by which target audiences are addressed can be expanded to include interaction tools, which support the interactions between people and between people and SEM models (Mayer, 2008).

In order to widen the accessibility of SEM studies through interaction tools, it is important to consider the following aspects: (1) system complexity and (2) actors’ dimension. Regarding system complexity, SEM studies tend to represent systems with complex structures and dynamics, which have been found to be difficult for humans to understand, especially if they include accumulations, feedback loops, and nonlinear behavior (Dieleman & Huisingh, 2006; Sterman, 2000). Regarding the actors’ dimension, SEM studies usually neither address the actors of the represented systems nor their interests, values, and perspectives. The consideration of the actors is particularly important because they manage and transform the underlying physical system and they tend to have different and conflicting interests (Wiek et al., 2006).

So far, SEM studies have addressed the aspects above by using different approaches and tools. For example, interactive visualizations and simulators have been used to improve the communication and interpretation of the results of studies representing complex systems (Riehmann et al., 2005; Vivanco et al., 2018). However, it has been found that learning through interactive visualizations and simulators mainly occurs in guided aftermath discussions (Dieleman & Huisingh, 2006; Milrad et al., 2003). The learning triggered through interactive visualizations and simulators normally remains on the cognitive level, and although cognitive learning can trigger long-lasting memories, it is only when combined with affective learning that users are more likely to question and critically reflect on their attitudes (Bornemann et al., 2020; Gatti et al., 2019).

One approach to include the actors’ dimension is the integration of SEM models with other models and frameworks. Examples of such models and frameworks are (1) structural agent analysis (SAA) models, (2) action-in-context (AiC) frameworks, (3) multi-attribute utility theory (MAUT), and (4) agent-based models (ABMs). The SAA models provide a framework to analyze how social structures and stakeholder’s decisions affect material flow management (Binder, 2007a, 2007b). The AiC framework allows the modeler to explicitly connect the physical system and their social driving forces, such as chains of actors, actions, and decision-making mechanisms (Hobbes et al., 2007) to identify pivotal actors and relevant policies to control the system. MAUT is an assessment method used to measure the attractiveness of different scenarios based on a set of environmental, economic, and social attributes, or criteria (Dyer, 2005; Rochat et al., 2013). ABMs represent decision-making processes via agents, who, restricted by certain attributes and behavior rules, make decisions and interact with the system and/or other agents (Schwarzer et al., 2018). The mentioned approaches cover the actors’ dimension mostly considering actors as objects, thus they are highly useful to include different actors’ rationale and perspectives. Nevertheless, they can be complemented by approaches in which actors are explored in a subject and participatory way such as participatory modeling (Anand et al., 2016).

Participatory modeling is a model development method characterized by the involvement of the stakeholders or actors in the actual modeling process (Voinov et al., 2016). The use of participatory processes provides actors the opportunity to design and describe the model form an early stage of development, thereby increasing transparency and trust on the models (Davies et al., 2015).

The approaches and tools mentioned in the previous paragraphs have been found successful and useful to address some of the relevant aspects related to the accessibility of SEM models, the communication and understanding of systems with complex dynamics, and the consideration of the actors’ dimension. However, none of the presented approaches tackle these aspects through interaction tools that allow a wide audience to experience and be the subjects in SEM systems, while facilitating learning about complex systems by triggering long-lasting memories and critical reflections on attitudes. SGs fall under the description of such interaction tools, and thus, we introduce them in the next section.

## MAIN FEATURES AND APPLICATIONS OF SIMULATION GAMES

In this section, we describe the main features, applications and purposes of SGs, and we present existing SGs in the field of industrial ecology (IE) and the simulation models commonly used in SGs addressing environmental issues.

SGs are games used to reproduce dynamic real-world phenomena and thereby allowing players to experience a system and its evolution over time, which often requires the integration of aspects from different disciplines (Capaul & Ulrich, 2010; Duke et al., 2008). While the field of simulation and gaming originally emerged from war-gaming (Mayer, 2009; Smith, 2009), SGs are nowadays widely used in areas such as business, health care, urban planning, and environmental issues (Klabbers, 2014; Kriz et al., 2014; Meijer & Smeds, 2014; Zürn et al., 2014).

Typically, SGs are applied in facilitated workshops that consist of three phases: the introduction, the simulation, and the debriefing phase (Duke & Geurts, 2004). During the introduction, the players are familiarized with the scenario and the procedures of the game. In the simulation phase, players play the game and thereby gain experiences in an immersive setting of social interaction. In the debriefing, the players reflect on the game experiences by using a structured process (see, e.g., Petranek et al., 1992 for the commonly used 4E method) and transfer relevant findings from the game to their everyday contexts. The debriefing phase is crucial for achieving workshop objectives and can be customized to the specific contexts.

Methodologically, the field of simulation and gaming draws from several sources, such as role-play, simulation, case studies, and games (Capaul & Ulrich, 2010). Role-play elements enable participants to adopt actor-specific perspectives and to understand the actor’s rationale and objectives. Simulation stands for a dynamic replication of real processes reduced to the essentials, which allows, amongst others, to compress time. Game elements such as goals, rules, and scores create an intensive, immersive game experience that can be further enriched by case study elements with situations from the everyday life or professional field of action. SGs can trigger specific signals or stimuli, for example, by images and emotions, allowing players to undergo an immersion process that exposes them to essential system elements enabling insights in to systemic relationships (Caluwé et al., 2008; Ulrich & Zemp, 2019). In addition, SGs with a role-play provide a set-up to experience relevant social interactions within a system, for example, by means of negotiations and discussions with other roles (Rodela et al., 2019; Ulrich & Zemp, 2019).

SGs can be developed and applied for various target audiences, such as students, professionals, or the general public (Gerber et al., 2021). Similarly, SGs can be used for various purposes, which include education, training, strategic planning, policy testing, stakeholder involvement, and data collection. In education and training, games can foster a paradigm shift from a passive (pure reflection) to an active learning process (Carreira et al., 2017) involving multi-modal learning (Cushman-Roisin et al., 1999; Dieleman & Huisingh, 2006). Regarding strategic planning and policy testing, SGs provide a “safe environment” to deal with systemic problems that include uncertainty and risk (Duke & Geurts, 2004). Concerning stakeholder involvement, SGs are used to include stakeholders in a participative manner, for example, to test and generate new ideas (Tóth, 2015). Furthermore, SGs can be applied as tools to gather data regarding stakeholders’ decisions in a research context (Kopainsky et al., 2019; Lebel et al., 2016).

For the development of SGs, several approaches are available, ranging from well-described and structured processes (see, e.g., Duke & Geurts, 2004) to stakeholder focused approaches (see, e.g., Étienne, 2013) to more open and simple guidelines (see, e.g., Jones, 1998).

### Simulation models in existing SGs addressing environmental issues

Some SGs addressing environmental issues include models that simulate the consequences of players’ decisions. These models are usually based on approaches such as (1) life cycle assessment (LCA), (2) integrated assessment models (IAM), and (3) system dynamics (SD).

A nonexhaustive search revealed that LCA models have been used in few instances, such as the board game developed by Cushman-Roisin et al. (1999), where the LCA model illustrates the supply chain of the US automobile industry. IAMs have been more widely used in SGs, for example, in the game developed by Valkering et al. (2012), which portrays the management of a typical Dutch river stretch under unexpected events such as flooding, or the game “Climate-Change Policy Exercise” (Parson, 1996), which contains an IAM representing global climate change. Similarly, SD models have been encountered in several cases, such as the “World Climate” game, which represents the UN climate change negotiations through an in-person role-play experience (Climate Interactive, n.d.; Sterman et al., 2014). Another example is the “Global sustainability crossroads,” a game where players can design and test climate-change mitigation strategies (Capellán-Pérez et al., 2019).

Within the field of industrial ecology (IE), SGs addressing environmental issues are rather sparse. We only found two SGs: (1) the industrial business symbiosis game, which simulates the strategic business dynamics of industrial symbiosis using mathematical models to compute waste flows and economic costs and benefits (Fraccascia et al., 2021), and (2) the board game of Cushman-Roisin et al. (1999) presented above.

While the models found in SGs addressing environmental issues tend to provide limited descriptions of the physical system, SEM models describe the physical aspects of the economy based on well-established physical principles, such as mass- and energy-balance principles, which results in highly robust representations of physical systems (Baccini & Brunner, 2012; Fischer-Kowalski, 1998; Fischer-Kowalski & Amann, 2001; Fischer-Kowalski & Haberl, 1998; Pauliuk & Müller, 2014). To the authors’ knowledge, no SG has yet been developed based on an SEM model. Thus in the next section, we present a novel case study, in which a SG based on an SEM model was developed.

## CASE STUDY: postfossilCities

In this section, we present the development and application of the postfossilCities SG, which includes an SEM model. This case study illustrates the link between SEM models and SGs and it serves as the basis to later discuss the benefits and challenges of such link.

### Game description

The postfossilCities SG has been developed in the context of a research project by a multidisciplinary team that included game developers, software developers, and SEM researchers. The game provides an experimental space where players can explore the transformation to a post-fossil future in Switzerland, that is, a future that is not relying on fossil resources. The game allows players to test different climate change mitigation measures in a negotiation setting. By playing the game, players learn about (1) different measures and their effectiveness in reducing GHG emissions, (2) the urgency to act, and (3) the systemic dimension of climate change mitigation. Furthermore, by taking on one of seven game roles, players get to experience different—possibly unfamiliar—perspectives, and are encouraged to think in strategic alliances. The game is targeted at current and future decision-makers, but also at the general public, especially to individuals motivated to engage in climate change mitigation.

**FIGURE 1:**
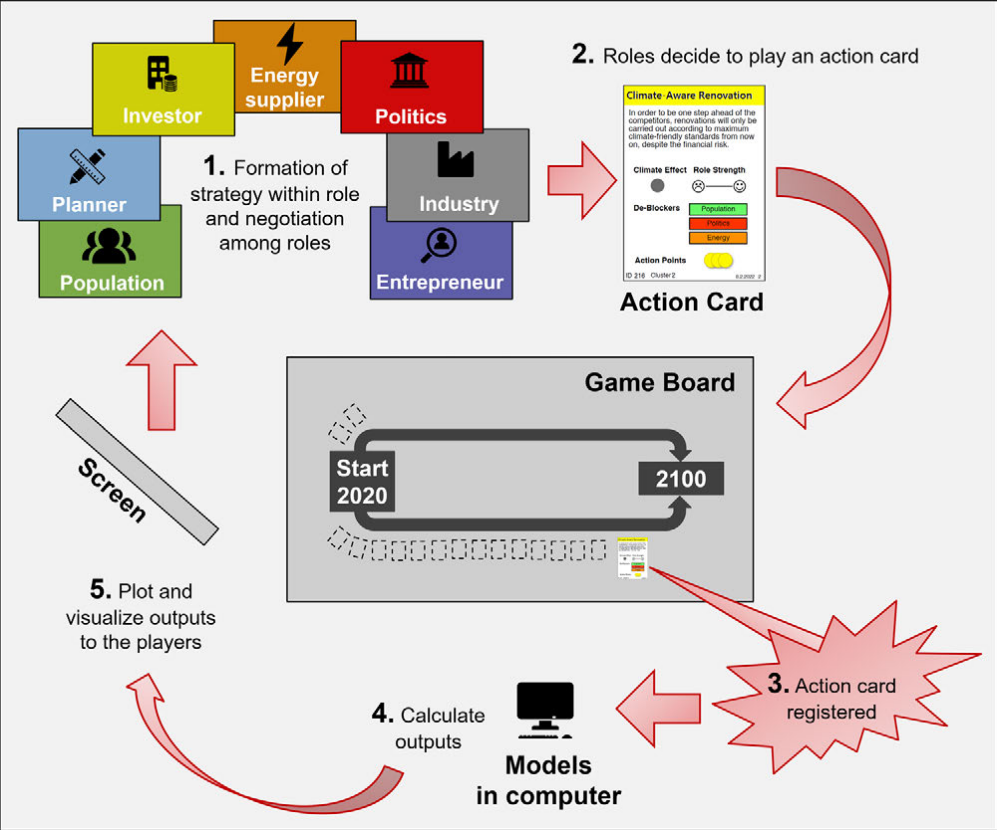
Game flow and main elements of the postfossilCities simulation games.

The game is applied in workshops, which last 3−6 h and are facilitated by one to two trained facilitators. It features seven roles, each of which is played by one to four players. Each role has its own, role-specific goal, and at the same time works toward the common goal of reducing the GHG emissions to net zero without exceeding the available carbon budget (Friedlingstein et al., 2020). To pursue their goals, players are given a set of “action cards” that represent measures they can implement. If an action card is played, the measure is implemented and the consequences are evaluated in different models, including an SEM model, that provide immediate feedback regarding GHG emissions, the carbon budget, and the role-specific goal (see the game flow in Figure 1). The game is played in rounds, where each round represents a third of a decade and includes strategy formation, negotiations, and evaluation of the decisions. The amount of implementable measures is limited by action points, an in-game currency representing resources such as time, money, or efforts that are used to implement measures. The number of action points for each role is updated after each round based on the achievement of the role-specific goals. To enhance the effect of a specific action card on GHG emission reduction and on the achievement of role-specific goals, other roles can play measures addressing the same topic (i.e., by playing cards from the same “cluster”).

### Game development and application

The postfossilCities SG has been developed by following the approach described by Duke and Geurts (2004), which included several steps and milestones. A first step was to define key characteristics of the game, such as the target audience, the problem addressed, the objectives, the duration, and the number of players. The next step was to conduct an analysis of the social and physical system, in which we identified relevant actors, collected pertinent information and data, and mapped relevant concepts and theories. Ultimately, the analysis was structured into a graphical visualization, which is shown in Supporting Information S1. The components of the analysis were then systematically translated into game elements, such as roles, rules, indicators, and models. Based on this translation, the format of the game was conceptualized, that is, the physical environment and the process through which the exercise is presented to the players. The next step was to implement the concepts in a prototype, which was continuously improved in an iterative process of testing and developing that lasted around 1.5 years.

The game has been applied in 12 on-site and virtual workshops in different settings, including public administrations, university courses, conferences, and public workshops. Further information on game applications is available at: www.postfossilCities.ch

### Models

**FIGURE 2:**
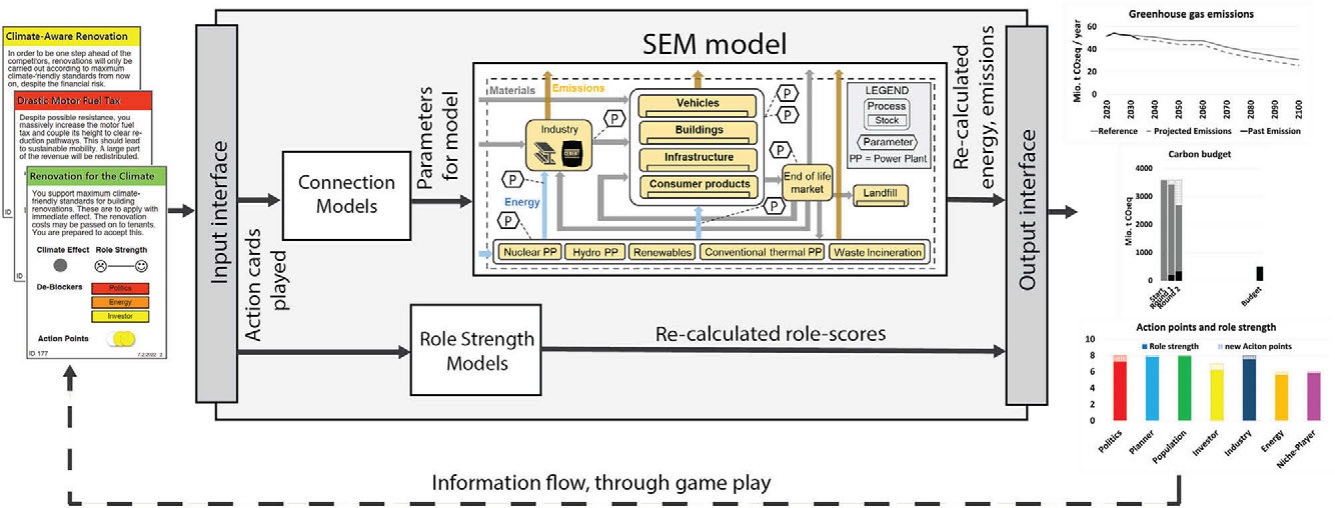
Overview of measures (action cards), models, connections, and player feedback.

The postfossilCities SG makes use of different simulation models to evaluate players’ decisions in terms of the common goal to reduce GHG emissions and the role-specific goals. Figure 2 provides an overview of the models and their connections. An SEM model including the building, transportation, energy, and industry sectors is used to calculate energy use and GHG emissions. In the development of the postfossilCities SG, we considered it important that the decisions presented to the players are based on their everyday situations and public discourses. Consequently, we refrained from the idea that players could directly manipulate the parameters of the SEM model. Instead, we implemented simple models (connection models) that translate measures described on “action cards” into changes of the SEM model parameters. Thus, the connection models link the social and physical dimensions. In the game, the social dimension is represented via the role-play and the “action cards,” whereas the physical dimension is represented by the SEM model. Furthermore, we developed so called “role strength” models to calculate the achievement of the role-specific goals.

Technically, both connection and role-strength models function in a similar way: based on the current state of implemented measures (i.e., played action cards) they select modification factors from a defined list, which are applied to the relevant SEM model parameters (in the case of the connection models) and the role-strength indicator of the previous round (in the case of the role-strength model). While the modification factors of the connection models were—where possible—estimated from the available literature to ensure robustness of model outputs, the modification factors of the role-strength models were chosen according to the rationale that the more roles support a particular measure, the higher the role-specific reward, which should trigger relevant in-game dynamics and experiences but is not based on further scientific evidence or theory.

Figure 2 shows the model outputs provided to the players, that is, the past and projected development of GHG emission, the state of GHG emissions in relation to the carbon budget, and the state of the role-strength indicator.

### Evaluation

The application of the postfossilCities SG was evaluated by means of (i) discussions with the players, which resulted in ample anecdotal evidence, and—for explorative purpose—(ii) a survey with pre- and post-game questionnaires with a small sample of participants (the questionnaires and their results are shown in Supporting Information S1 and S2). The questionnaires were answered by the 42 participants of two workshops; however, only 16 answered both the pre- and post-game questionnaires. The “discussions with the players” took place with the 42 participants of the two workshops and with the participants of the 10 additional application workshops. We acknowledge that the survey sample is very small, and as such the summary of the main outcomes are only indicative of the sample, thus rendering generalization difficult. To generalize our findings, more sample data would be required.

Based on the above explained evaluations, the game was regarded as an effective tool to learn about transitions toward a fossil-free society, in particular, regarding (1) the importance of cooperation and alignment between different actors, (2) the systemic aspect of the transition, (3) different climate-change mitigation measures and their priority, and (4) the time dimension and urgency for action. The game was also found to challenge players’ mental models, for example, in terms of time delays related to the effects of measures. Furthermore, the game workshops allowed players to translate the insights from the game to their everyday private and public contexts. For example, players mentioned their willingness to fly less or eat less meat.

The use of questionnaires for data collection has limitations. In the case of the questionnaires used in the postfossilCities SG, some relevant limitations included: (1) the lack of a mid- or long-term evaluation of the game impacts on players, (2) no in-depth analysis of the specific impacts on players’ knowledge and behavior, and (3) the inflexibility of the response options, that is, multiple-choice questions limit participants’ answers to the given options. Despite all the mentioned limitations, we chose questionnaires as opposed to other data collection methods because of the easiness of comparing players’ behavior and knowledge before and after the game, the possibility to conduct the surveys close in time to the game-play session, and the resource effectiveness of the method (including time and cost resources), among others.

## EXPLORING THE LINK BETWEEN SEM MODELS AND SGS

In this section, we discuss the link between SEM models and SGs by reflecting on potential benefits and challenges. Our reflections are based on the postfossilCities SG, experience with other existing SGs, and the literature. Regarding the case study, both the process of developing the SG and the outputs and evaluations from various SG workshops were used to inform the discussion below.

### Benefits of linking SEM models and SGs

The link between SEM models and SGs can add valuable benefits to both the SEM and the SGs communities. First, we explore the benefits from the SEM perspective, followed by the benefits from the SGs perspective.

#### Benefits of using SGs based on SEM models

SGs have been found to be successful in supporting problem-solving processes for complex, systemic challenges, such as climate change (Gerber et al., 2021; Rooney-Varga et al., 2020), which might be explained by the following strengths of SGs: (1) targeted abstraction, (2) Gestalt communication, (3) safe experimentation in a protected environment, (4) exploration of intended, unintended, and side effects, (5) re-scaled timelines, that is, compress long time periods, and (6) translate simulated concepts to the everyday life language of the players (Ulrich, 2006; Ulrich & Zemp, 2019). Next, we explore how the mentioned strengths of SGs can bring benefits to SEM research, in particular, regarding the communication and understanding of systems with complex dynamics and the consideration of actors’ dimension.

*Targeted abstraction* is about bringing specific system elements to the focus of players and thereby allowing players to directly experience essential aspects of systems (Ulrich & Zemp, 2019). Highly abstract games, for example, allow insights into general system properties, while highly contextualized games can be useful to gain insights into a specific problem. *Gestalt communication* refers to the ability of games to provide an integrative perspective on a phenomenon, for example, through the parallel use of different forms of communication such as reading, writing, experiencing, listening, talking, or doing (Duke & Geurts, 2004). Both strengths of SGs are useful to make system complexity of SEM models “tangible” in an experimental manner. In particular, SGs can draw the attention of players to specific elements of the systems represented in SEM models, for example, car fleet in a city, and provide certain stimuli, for example, large decrease in the car fleet, to facilitate the understanding of systemic connections and complex system dynamics typical of SEM studies. Furthermore, targeted abstraction and Gestalt communication make SGs useful tools to ease the comprehension of complex SEM systems because learners acquire new knowledge through cognitive and affective learning (Despeisse, 2018; Gatti et al., 2019). In the case of SGs, cognitive learning usually takes places via experiential- and action-learning processes, in which learning occurs through reflection upon “doing” (Barth et al., 2007; Figueiró & Raufflet, 2015; Springett, 2005). For example, in the postfossilCities SG, players reported learnings about the systemic character of their actions and of the transition toward a post-fossil future. Affective learning refers to the values, opinions, attitudes, behaviors, and emotions of the learners (Gatti et al., 2019; Shephard, 2008). For example, in the postfossilCities SG, players felt emotions such as frustration due to actions not decreasing GHG emissions as they expected. Unsurprisingly, learning through SGs have been found to be more effective than lecture-driven learning, because they can trigger longer-lasting memories and critical reflections of one’s own attitudes (Bornemann et al., 2020; Gatti et al., 2019; Lohmann, 2020; van Eck, 2006; Wouters et al., 2013). Ultimately, by providing the “right” level of abstraction and applying Gestalt communication principles, SGs provide environments where players engage in an experiential and immersive process through which complex SEM systems and their dynamics can be understood more easily (Caluwé et al., 2008; Rodela et al., 2019; Ulrich & Zemp, 2019).

In SGs, players can *safely experiment in protected environments*, which means that players can test uncommon and radical strategies in a set-up that is free of the usual sanctions or consequences of the world outside the game (Caluwé et al., 2008). In SGs with role-play, players take on different roles and thereby explore actors’ perspectives in a subject and active way, allowing them to (1) explore different and potentially conflicting interests of actors and (2) gain new perspectives on problems, potential solutions, and interactions (Batten, 2009; Breuer, 2008; Chen & Martin, 2015; Dieleman & Huisingh, 2006; Peters & van de Westelaken, 2008; Schwägele, 2014; Wesselow & Stoll-Kleemann, 2018). In SGs, players can also explore, experience, and feel *intended, unintended, and side effects* of their decisions and actions, as SG provide immediate feedback on players’ actions, for example, based on SEM models. In SGs based on SEM models, the actors’ dimension represented in the role-play can be closely connected to the physical systems represented in SEM models. Thus, SGs based on SEM models can make the exploration of the effects of specific actions on both the physical and social dimensions highly accessible to wider audiences.

In addition, SGs are also capable of *re-scaling time periods*, for example, by compressing long time periods into a game setting of a few minutes or hours. In this way, the time periods become manageable and allow participants to experience long-term developments. This is especially important for exploring the transformation of SEM systems in regard to strategies for climate change mitigation or circular economy as such transformations occur in a time span of several decades (Müller et al., 2013). Thus, SGs allow players to experience potential difficulties and challenges related to time, for example, effects with long time delays.

Finally, the game setting allows to *translate the simulated concepts to the everyday life language of the players*, by creating a game environment with familiar terms and concepts. While concepts such as “useful floor area per capita” are commonly used among SEM scholars, they may not be intuitive for many audiences, for example, general public. In a game, such concepts can be presented by using descriptions that are familiar to wider audiences, for example, “size of flats.” Expressions close to the players’ everyday life situation increase the relevance and connectivity of the game experience.

#### Benefits of using SEM models in SGs

As seen in Section 3, existing SGs addressing environmental issues are commonly based on a wide variety of simulation models. These simulation models often include limited representations of the physical economy, hindering the explanation of the nexus between built environment, circular economy, and climate change mitigation. For example, in IAMs, the relations and dependencies between materials and energy throughput, stocks and services tend to be omitted or poorly represented, which leads to material stocks being either not included or inconsistently covered (Pauliuk & Müller, 2014). In the case of SD models, they often cover both, the physical and the social systems, but are in some cases less sophisticated in the physical description. As opposed to the examples above, SEM models allow to define the physical world by a system of stocks and flows of energy and materials based on well-established physical principles, that is, mass- and energy-balance conservation (Baccini & Bader, 1996; Fischer-Kowalski, 1998; Fischer-Kowalski & Amann, 2001; Fischer-Kowalski & Haberl, 1998; Pauliuk & Müller, 2014). In SEM models, these principles are used to define the physical systems over space and time, for example, with dynamic models covering a time span of several centuries (Roca-Puigròs et al., 2020). Furthermore, the physical systems in SEM models can be represented in high detail by defining multiple layers for different physical components and tracking them over time (Løvik et al., 2014). Thus, the benefit of using SEM models is that they are robust at representing physical systems in a mass- and energy-balance consistent way and therefore, they can contribute to making SGs more robust.

As exposed previously, the actors’ dimension or social realm is only roughly covered in SEM models. Thus, as opposed to SD models, if the actors’ dimension is to be included, SEM models need to be expanded to include the social realm, for example, by using SGs. By linking SEM models to SGs, one can experience both the challenges of the social realm, for example, negotiations among actors with different interests, and of the physical realm, for example, time delays of certain measures. In this way, the physical world governed by physical laws, creates boundary conditions to the social world, which is governed by values, interests, and social norms. Thus, SGs based on SEM models can provide highly realistic system exploration set-ups because they are constrained by the physical boundary conditions of SEM models.

### Challenges of linking SEM models and SGs

Besides the exposed benefits, the development of SGs based on SEM models also comes with challenges and critical aspects, which should be considered when starting a game development process. Next, we describe and discuss such challenges and critical aspects.

#### Integration of different disciplines

The development of SGs requires the involvement of approaches from different disciplines such as simulation and gaming, mathematical modeling, social sciences, and computer science. The final constellation of disciplines depends on the specific game’s purpose, design, and application. Jointly developing an artifact implies that it is necessary to fully integrate the different disciplines in the course of game development into a common artefact, that is, the game, which means that a certain understanding across disciplines is required to avoid misunderstandings. The development of a common “language” can help to avoid misunderstandings (Müller et al., 2005).

#### Resource intensity

Substantial resources in terms of time, effort, and finances may be required to develop SGs. An example of a particularly time-consuming step in game development is the iterative development of game prototypes trough testing and refining. In the development process of the postfossilCities SG, this step took more than a year and has been found crucial to ensure playability and relevance of the game. Thus, resources should be considered and addressed in an early stage of the process, for example, in the outset of the project, so that the project timeline can be planned accordingly. In addition, early definition of the interfaces between the disciplines involved in the SG development process can effectively reduce resource use.

#### Game settings and actors

For a successful game development, it is crucial to define the settings of the game application and to identify relevant stakeholders. The settings include aspects such as the target audience, number of players, game duration, and topic. Game methodologies usually guide practitioners through the steps of defining game settings and actors; however, they may not emphasize the importance of such steps. Thus, it is recommendable to undertake such steps with great care on an early point of the game development process because they are important for later development steps.

#### Integration of SEM models in SGs

There is a gap between players’ world and SEM models, which needs to be addressed, as players are often unfamiliar with parameter names, such as typesplit of new passenger cars. Different approaches are available to address this gap. The approach used in the postfossilCities SG, consists of simple mathematical models, which link the parameters in SEM models and the game. To ease this drawback, a generic framework formulation of these additional models could be published for re-use.

#### Combining analytical and creative skills

The development of SGs based on SEM models requires both analytical and creative skills. Analytical skills are crucial to ensure a solid scientific foundation, which is important for the credibility and value of the SG, whereas creative skills are important to provide the right designs, visualizations, sceneries, and environments to motivate and engage players, and ultimately trigger an immersion process into the game’s topic.

#### Balancing complexity and simplification

During the game development process, there is a balance to be found between presenting the complexity of SEM systems and simplifying such complexity by choosing relevant elements that allow players to comprehend the systems. In this balance, it is important to consider aspects that trigger player motivation and engagement, as well as the planned duration of the game and the target audience. In the postfossilCities SG, it was necessary to develop and test several game prototypes to find the right balance, especially to provide players with an adequate amount of information.

## FINAL REMARKS

Linking SEM models and SGs can bring valuable benefits, such as making SEM research more accessible to wider audiences, considering the actors’ dimension of physical systems and contributing to the robustness of SGs by having mass- and energy-balance consistent representations of physical systems. In addition, the link of SEM models and SGs can trigger further potentials by targeting the game to specific purposes. For example, SGs can be developed for educational purposes, which can be used to teach IE concepts such as waste prevention strategies to a wider audience. Another purpose of games is strategic planning and policy testing, where SGs could provide an experimental setting to explore certain interventions in an easy-to-understand, safe, and memorable way. Another example is SGs developed for data collection purposes that could be used, for example, to estimate the values of model parameters.

In order to facilitate the link between SEM models and SGs, and thus exploit the mentioned benefits, we suggest that the SEM modeling community (1) establish stronger connections to game-related communities, for example, International Simulation and Game Association (ISAGA); and to communities where SGs have long been used, to avoid recurrent mistakes, (2) use existing game development methods to facilitate game design and project planning, and (3) build a new section within the “International Society for Industrial Ecology” dedicated at outreach for promoting the use of approaches and tools such as SGs.

The field of SGs is evolving rapidly, in particular through the incorporation of new technologies, such as virtual reality. Although the future of SGs and the widespread adoption of such technologies is uncertain, these technologies could make the experience of playing SGs highly engaging and motivating, and could therefore bring additional potentials to the link between SEM models and SGs.

## Supplementary Information

**Supporting Information S1**: This supporting information provides (A) the visualization of the analysis performed during the development of the postfossilCities simulation game, (B) the survey, including pre- and post-Game Questionnaires, used to evaluate the postfossilCities simulation game, and (C) a summary of the results of the survey, including the main qualitative and quantitative results of the Questionnaires.

**Supporting Information S2**: This supporting information provides the complete results of the survey, including pre- and post-game questionnaires, of two game workshops. The results are given in an anonymous manner to protect the identity of the participants.

## Data Availability

The data that supports the findings of this study are available in the supporting information of this article.
