# Supplementary material for: Linking socio-economic metabolism models and simulation games: Reflections on benefits and challenges
Source: J Ind Ecol. 2024 Feb 2;28(2):182–93. doi: 10.1111/jiec.13462 (PMC13083430; doi:10.1111/jiec.13462)
Supplement: Supplementary file 1 — Supporting Information S1: This supporting information provides (A) the visualization of the analysis performed during the development of the postfossilCities simulation game, (B) the survey, including pre- and post-Game Questionnaires, used to evaluate the postfossilCities simulation game, and (C) a summary of the results of the survey, including the main qualitative and quantitative results of the Questionnaires. [file 44498_2024_2802001_MOESM1_ESM.docx]

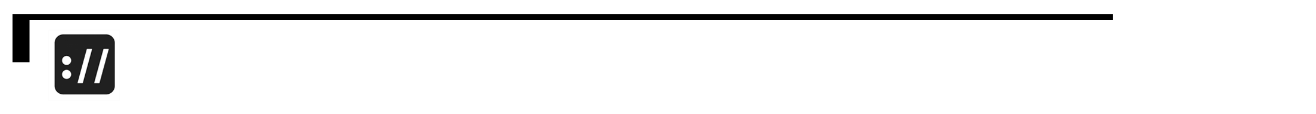


SUPPORTING INFORMATION FOR:

Roca-Puigròs, M., Gerber, A., Ulrich, M., Reich, M. Y., Müller, D. B. & Wäger, P. (2024) Linking Socio-Economic Metabolism models and Simulation Games: Reflections on benefits and challenges. *Journal of Industrial Ecology.*


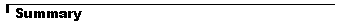


This supporting information provides (A) the visualization of the analysis performed during the development of the postfossilCities simulation game, (B) the survey, including pre- and post-Game Questionnaires, used to evaluate the postfossilCities simulation game, and (C) a summary of the results of the survey, including the main qualitative and quantitative results of the Questionnaires.


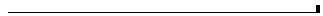


# A – Game development: visualization of the analysis

One of the steps of the game development processes according to Duke and Geurts (2004) consists in visualizing the system analysis of the problem. **Figure A1** shows the graphical visualization of key information used to develop the postfossilCities simulation game (SG). The information has been allocated to the following categories: current state, boundaries (including environmental boundaries), actors, socio-economic metabolism, transformation (including points of departure, theories of transformation, options for the transition, examples for operationalizing the transition, windows of opportunities, and system mechanisms), and possible future states.


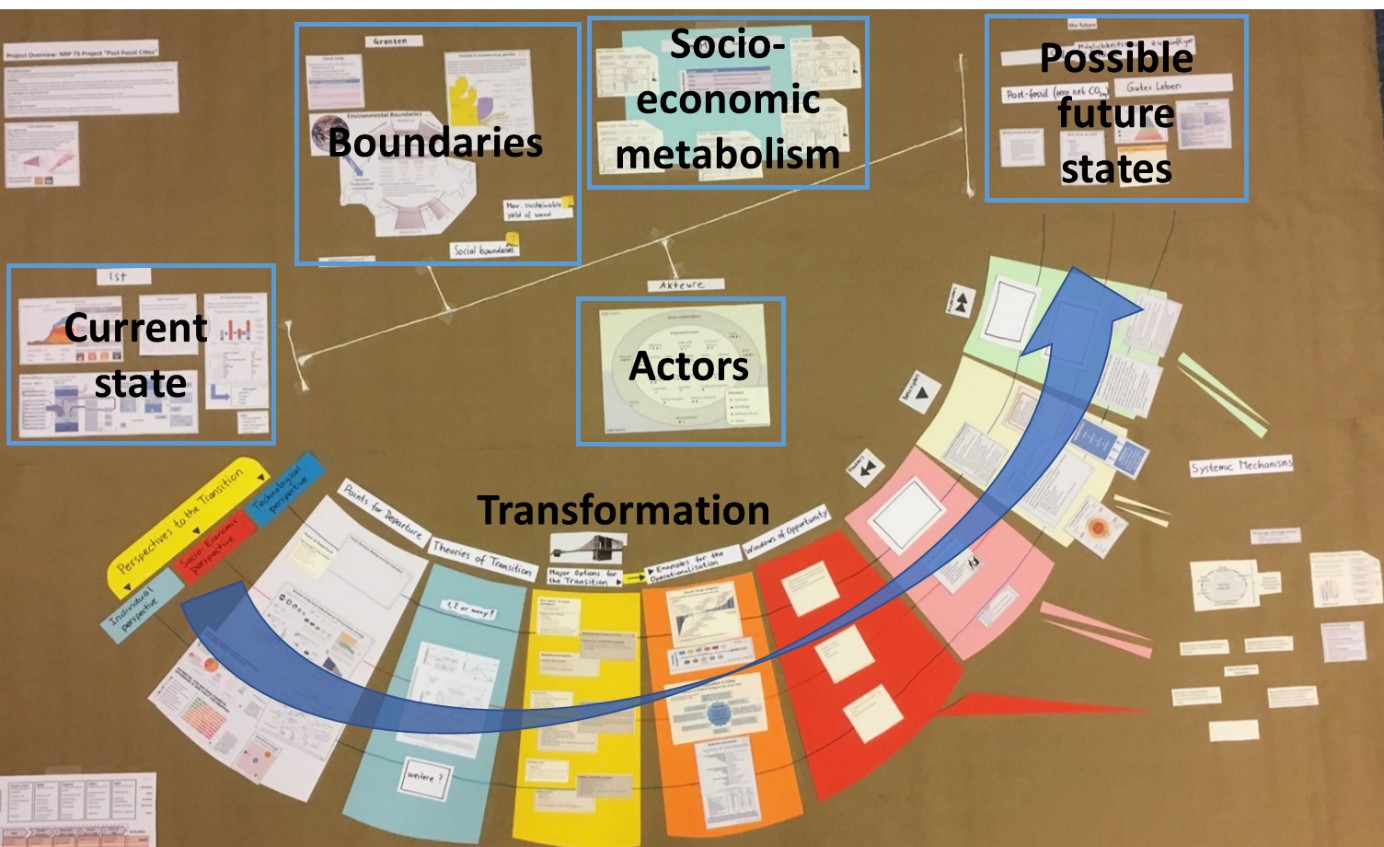


**Figure A1** A visual representation of key information used for the development of the postfossilCities simulation game. Note that the SEM model is one element of the schematic.

# B – Survey with the pre- and post-game questionnaires

| **Pre-Game Questionnaire** |
| --- |
| **Q1** How concerned are you about climate change?  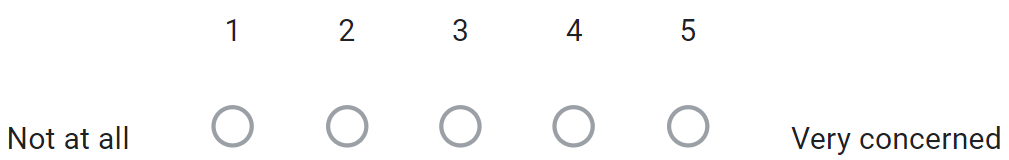 |
| **Q2** How well do you think you understand the transition towards a climate-friendly society?  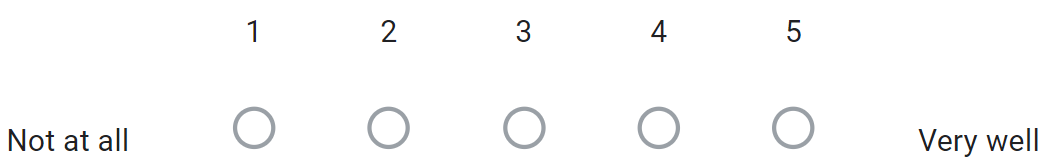 |
| **Q3** What actions/policies are most important to reduce emissions in a big city? Specify 3-5 actions. Prioritize them writing a number in brackets, 1 = highest priority. State the easiness of implementing such actions by specifying in between brackets: easy, moderate, difficult. See an example below.   - *Action description (1) (moderate)* - *Action description (3) (difficult)* - *Action description (2) (easy)* |
| **Q4** Do you consider your lifestyle sustainable?  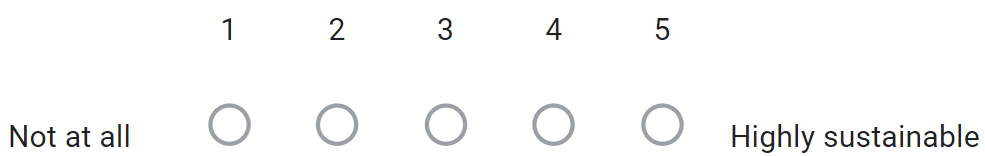 |
| **Q5** What do you hope to learn in the game? |

| **Post-Game Questionnaire** |
| --- |
| **Q1** What did you learn in the game? |
| **Q2** How concerned are you about climate change?  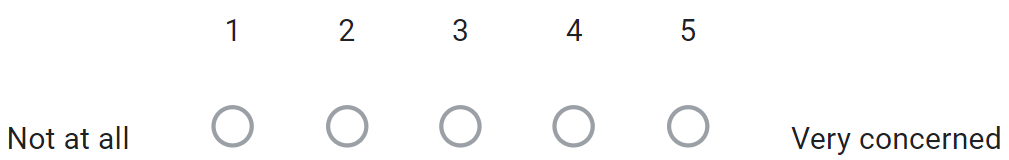 |
| **Q3** How well do you think you understand the transition towards a climate-friendly society?  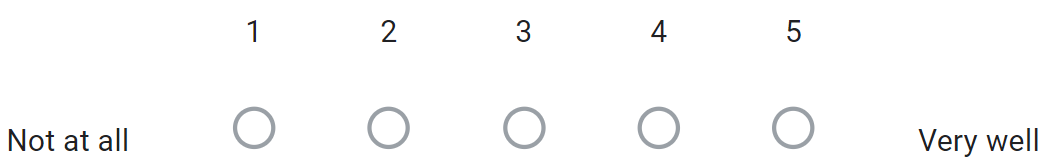 |
| **Q4** What actions/policies are most important to reduce emissions in a big city? Specify 3-5 actions. Prioritize them writing a number in brackets, 1 = highest priority. State the easiness of implementing such actions by specifying in between brackets: easy, moderate, difficult. See an example below.   - *Action description (1) (moderate)* - *Action description (3) (difficult)* - *Action description (2) (easy)* |
| **Q5** Do you consider your lifestyle sustainable?  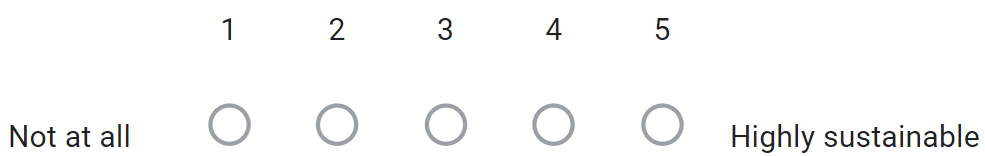 |
| **Q6** Is there something you are willing to change regarding your lifestyle? |
| **Q7** Do you think your knowledge on transitions towards a climate-friendly society improve?  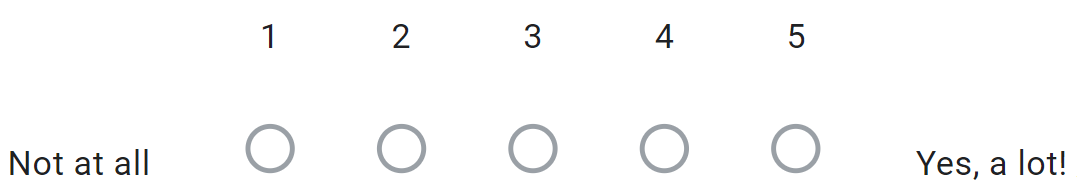 |
| **Q8** What is the most important take-home message from the game? |

# C – Results of the survey

The postfossilCities simulation game (SG) was evaluated by means of a survey, including pre- and post-game questionnaires (see **Appendix B**). The questionnaires contain quantitative and qualitative questions. The answers to the quantitative questions, which were included in both the pre- and the post-game questionnaires, were assessed with a paired t-test to check for statistically significant differences. The questionnaires were answered by a total of 42 participants; however, only 16 answered both the pre- and post-game questionnaires. The sample size of participants who answered both questionnaires is rather small, and thus, the power of the statistical test should not be regarded as high.

**
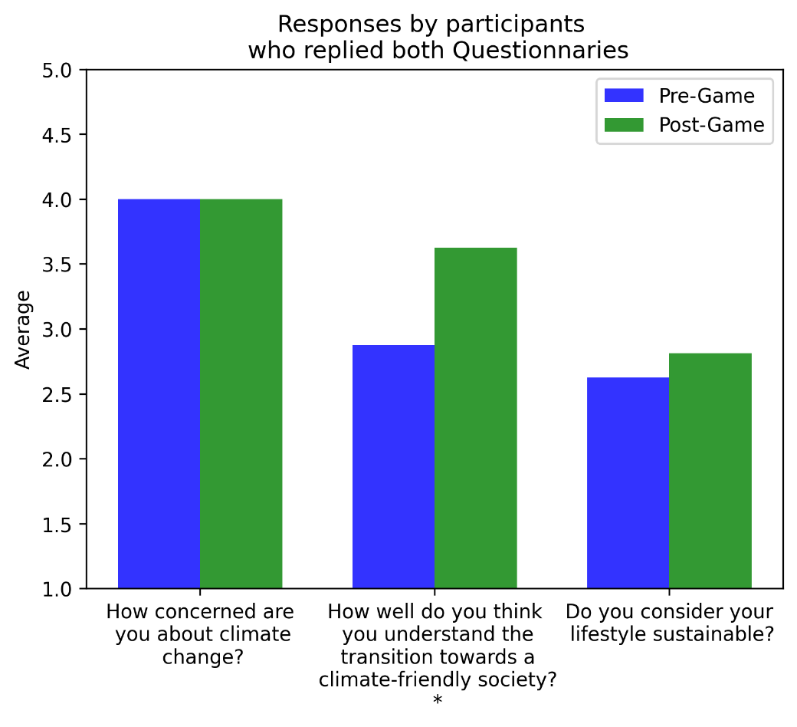
**

**Figure C1** Results on the quantitative questions included in both the pre- and post-game questionnaires, with 1 being the lowest score and 5 the highest, and the results of the statistical pared t-test. The answers to the questions showing statistically significant differences between pre- and post-game results are marked with an asterisk (*).

**Figure C1** presents the results on the quantitative questions included in both the pre- and post-game questionnaires and the results of the statistical paired t-test. The results indicate that players' concern about climate change and their consideration of having a sustainable lifestyle did not significantly change after playing the game. However, the participants reported a significant increase in the understanding of transitions towards a climate-friendly society. The numerical data plotted in **Figure C1** is provided in **Table C1**. Furthermore, in the post-game questionnaire, the participants reported that their knowledge on transitions towards a climate-friendly society after playing the game slightly improved. Regarding the qualitative questions, most participants prioritized the most important actions to reduce emissions differently before and after the game. While transportation-related actions were considered by far the most important before the game, after the game, building-related actions were regarded equally important and renewable energies-related actions gained in importance. After the game, the participants reported learnings in three main areas: (1) stakeholders cooperation, (2) measures to tackle climate change mitigation, and (3) systems perspective. Furthermore, after the game, most participants reported their willingness to change their lifestyle by, for example, flying less and eating less meat.

**Table C1** Numerical data plotted in Figure C1

| **Questions from the survey** | **Pre-Game** | **Post-Game** |
| --- | --- | --- |
| How concerned are you about climate change? | 4 | 4 |
| How well do you think you understand the transition towards a climate-friendly society? | 2.9 | 3.6 |
| Do you consider your lifestyle sustainable? | 2.6 | 2.8 |

# References

Duke, R., & Geurts, J. (2004). *Policy games for strategic management. Pathways into the unknown.* Amsterdam, The Netherlands: Dutch University Press.
